# Association of the Asn306Ser variant of the *SP4* transcription factor and an intronic variant in the β-subunit of transducin with digenic disease

**Published:** 2007-02-28

**Authors:** Yong-Qing Gao, Michael Danciger, Riza Köksal Özgül, Yekaterina E. Gribanova, Samuel G. Jacobson, Debora B. Farber

**Affiliations:** 1 Jules Stein Eye Institute, David Geffen School of Medicine at UCLA, Los Angeles, CA; 2 Biology Department, Loyola Marymount University, Los Angeles, CA; 3 Hacettepe University, Department of Molecular Biology, Beytepe-Ankara, Turkey; 4 Scheie Eye Institute, University of Pennsylvania, Philadelphia, PA; 5 Molecular Biology Institute, David Geffen School of Medicine at UCLA, Los Angeles, CA

## Abstract

**Purpose:**

SP4 is a transcription factor abundantly expressed in retina that binds to the GC promoter region of photoreceptor signal transduction genes. We have previously shown that SP4 may be involved in the transcriptional activation of these genes alone or together with other transcription factors such as SP1, neural retina leucine zipper protein (NRL), and cone-rod homeobox gene (CRX). Since mutations in *NRL* and *CRX* are involved in inherited retinal degenerations, *SP4* was considered a good candidate for mutation screening in patients with this type of diseases. The purpose of this work, therefore, was to investigate possible mutations in *SP4* in a cohort of patients affected with different forms of retinal degenerations.

**Methods:**

270 unrelated probands with various forms of retinal degeneration including autosomal dominant and autosomal recessive retinitis pigmentosa (RP), autosomal dominant and autosomal recessive cone-rod dystrophy (CRD), and Leber's congenital amaurosis (LCA), were screened for mutations in the *SP4* gene. Single strand conformation polymorphism (SSCP) analysis was performed on the six *SP4* gene exons including flanking regions followed by direct sequencing of SSCP variants.

**Results:**

Nine different sequence variants were found in 29 patients, four in introns and five in exons. Many of the probands were previously screened for mutations in the genes encoding the α-, β- and γ-subunits of rod-specific cGMP phosphodiesterase (*PDE6A*, *PDE6B*, *PDE6G*), the β-subunit of rod-specific transducin (*GNB1*), and peripherin/rds (*RDS*). One group of seven probands of Hispanic background that included five with arRP, one with RP of unknown inheritance (isolate) and 1 with arCRD carried an Asn306Ser mutation in *SP4*. Of the seven, the isolate case was homozygous and the other 6 heterozygous for the variant. Two arRP and the arCRD probands carried an additional intronic *GNB1* variant. DNA from the family members of the arCRD proband could not be obtained, but for the other two families, all affected members and none of the unaffected carried both the *SP4* Asn306Ser allele and the *GNB1* intronic variant.

**Conclusions:**

If mutations in *SP4* do cause retinal degenerative disease, their frequency would be low. While digenic disease with the *SP4* Asn306Ser and the *GNB1* intronic variant alleles has not been established, neither has it been ruled out. This leaves open the possibility of a cooperative involvement of *SP4* and *GNB1* in the normal function of the retina.

## Introduction

Transcription factors have been shown to play an important role not only in the biology of photoreceptors and other retinal cells, but also as sites of mutations causing degenerative disease. For example, mutations in *CRX* and *NRL* cause various forms of progressive retinal dystrophies [1-4]. The SP family of transcription factors (SP1-5) is formed by a group of proteins that selectively bind to the "GC box" in the promoter region of many genes [5-7] via three putative zinc finger domains of the C2H2 type [8]. Based on sequence similarities, SP1, SP3, and SP4 are more closely related to each other than to SP2 and SP5 [6,9] and the three have similar affinity for the GC box [10,11]. While SP1 and SP3 are ubiquitously expressed, SP4 is most abundant in the developing nervous system, particularly in the hippocampus [12] and retina [13], although it is also expressed in other tissues [10,14]. *Sp1* and *Sp3* knockout mice all die after E10 or at birth, respectively. *Sp4* knockout mice appear to develop normally to birth, but after birth, many pups die by P28 and those that survive are small and have other abnormalities [12]. In the last few years, we have studied extensively the SP4 transcription factor and have found that it is present in all retinal layers, interacts with CRX and NRL and activates transcription of several rod specific genes including *PDE6B* and *RHO*, encoding the β-subunit of cGMP-phosphodiesterase (β-PDE) and rod opsin, respectively [13,15,16]. Because of its specific involvement in transcription of rod genes and because of the history of transcription factor mutations causing retinal degeneration, we considered *SP4* a good candidate gene to screen for missense mutations in patients with various forms of retinal degeneration. To this end, we screened the 6 exons of the *SP4* gene in a group of 270 patients with various forms of retinal degeneration that had been screened previously for a number of photoreceptor genes [17-21]. Although we could not establish that mutations in the *SP4* gene cause retinal degenerative disease, neither could we rule this possibility out because the families of two patients in which an *SP4* missense mutation and a *GNB1* intron 2 variant were present, segregated with disease. Interestingly, the inherited retinal degeneration of the *Rd4* mouse is caused by an inversion of mouse chromosome 4, and the site of the telomeric breakpoint is precisely on intron 2 of the *Gnb1* gene [22].

## Methods

### Patients

270 patient probands of mixed ethnicities (56% European, 17% Asian, 13% Black, 14% Hispanic) were screened for variants in the six exons of the *SP4* gene, including 49 with autosomal dominant retinitis pigmentosa (adRP), 103 with autosomal recessive retinitis pigmentosa (arRP), 26 with autosomal dominant cone-rod dystrophy (adCRD), 52 with autosomal recessive cone-rod dystrophy (arCRD), and 40 with Leber's congenital amaurosis (LCA). Many of the above patients had been previously screened for mutations in the genes encoding rod- αPDE, βPDE, γPDE, rod β-transducin and RDS-peripherin. 95 controls with a similar ethnic distribution (58% European, 16% Asian, 13% Black, 13% Hispanic) were screened for each of the above genes and *SP4* as well. Written informed consent was obtained in compliance with the tenets of the declaration of Helsinki and with the approval of the office of Human Research Protection of the School of Medicine, University of California, Los Angeles.

### Polymerase chain reaction

Blood was drawn in 10-20 ml aliquots and DNA was extracted from the leukocytes by standard methods. Initial screening was done by SSCP as described previously [17-21]. The exons of *SP4* were amplified by polymerase chain reaction (PCR) directly from genomic DNAs with appropriate primers pairs. Each PCR amplicon included 50-150 nt of intronic flanking sequence on each side of the exon. The PCR protocol was 94 °C for 3 min followed by 30 cycles of 94 °C for 45 s, 55-60 °C for 45 s and 72 °C for 45 s, followed by 5 min at 72 °C. The sequences of primer pairs are presented in Table 1.

**Table 1:** Primer sequences used for *SP4* and *GNB1* screening.

Exon 1		Sp4	1F:	5’-TGTGCCAGCTACAGCCTCCT
		Sp4	1R:	5’-CCCCTTAAAGCATCTCAGCG
Exon 2		Sp4	2F:	5’-GCCCGCGCTCGCGGGTTTATGGAGATTA
		Sp4	2R:	5’-GGCGGCCAAAGGGCATAACTCCCTCTC
Exon 3	1	Sp4	3AF:	5’-GCAACTACTGGCCTCCACTA
		Sp4	3AR:	5’-GACCAGGGGTGGAAGAATT
	2	Sp4	3BF:	5’-ACTTGTTGCCTCCACTCCT
		Sp4	3BR:	5’-GTAGAGATGAACTACTAGTTGG
	3	Sp4	3CF:	5’-GCCCCCGCCGAAACTTCAGACAGTGGAAGGTC
		Sp4	3CR:	5’-CTGGCAAAGCTAGAGTCACT
	4	Sp4	3DF:	5’-GCCCCCGCCGAGGCTCAAGTTGTAACAACC
		Sp4	3DR:	5’-ATTGATCCTGTGCATTCTGC
	5	Sp4	3EF:	5’-CTACTGAGTCTGAAGCCCA
		Sp4	3ER:	5’-CGGGCGGGGGGTTGGGATAACCCAGCATTC
	6	Sp4	3FF:	5’-TTCAGGGCAAATCAGTTGGC
		Sp4	3FR:	5’-CAGAGGTCTACGTCAAACC
Exon 4		Sp4	4F:	5’-GCCATTAACCCCTTAGTTTTTG
		Sp4	4R:	5’-CCGGGCCCGGCCTGCTGTTTTATGCCTTCCAA
Exon 5		Sp4	5F:	5’-CAAACCTATTCAGAGAAAGCATC
		Sp4	5R:	5’-GCCTTCTCCTTCATTTGCAT
Exon 6		Sp4	6F:	5’-TATAATGTGACCAGAAGGGAG
		Sp4	6R:	5’-TAATCCACTTGACCAGCCCA
*GNB1*				
Exon 3		Gnb1	3F:	5’-GATCTCCTGACCTCGTGATCT
		Gnb1	3R:	5’-TAACATYCTAACCTGAAACTC

In each primer pair of this table F indicates the forward primer annealing to the DNA in the 5'-3' direction and R indicates the reverse primer, annealing to the DNA in the 3'-5' direction. Since exon three of *SP4* is long, six sets of primer pairs were used for its amplification.

### Single strand conformation polymorphism

Amplicons were separated by electrophoresis in 7% acrylamide gels and analyzed by standard P^32^ autoradiography or silver staining methods to reveal polymorphisms as described previously [17-21].

### Sequencing

Amplicons carrying polymorphisms were purified using the QIA QUICK PCR purification kit (Qiagen, Valencia, CA) and sequenced using the Dyenamic ET Terminator cycle sequencing kit (Amersham, Piscataway, NJ).

## Results

SSCP screening of the *SP4* gene showed 9 sequence variants in 29 patients, five present in exons (Table 2). The heterozygous Leu241Val and Pro286Ala missense variants, both present in exon 3 of arRP probands did not segregate with disease in the corresponding families. An Asn306Ser missense variant in exon 3 was present in both alleles of one isolate RP proband, in one allele of five arRP probands and in one arCRD proband. This missense variant was also present in 1/95 controls. Interestingly, although only 14% of the 270 patients and 13% of the 95 controls were Hispanic, all seven patients and the 1 control that carried Asn306Ser were Hispanic. Thus, 18.4% (7/38) of the Hispanic patients carried Asn306Ser while none of the other patients did, including 0/151 patients of European origin. The other 2 coding region variants were both silent. Neither was present in 95 controls. The remaining 4 intronic sequence variants were present in patients and absent from controls with the exception of -121 A to C which was present in one control (Table 2).

**Table 2:** Sequence variants detected in the screening of the *SP4* gene.

Variant	Exon/ Intron	Frequency in patients	Frequency in controls
Leu241Val	Exon 3	1/270	0/95
Pro286Ala	Exon 3	1/270	0/95
Asn306Ser	Exon 3	7/270	1/95
Ala276Ala	Exon 3	1/270	0/95
Gln451Gln	Exon 3	5/270	0/95
-121A→C	Intron 1	4/270	1/95
+52T→C	Intron 3	5/270	0/95
+70T→C	Intron 5	4/270	0/95
-37, 38Δ2C	Intron 2	5/270	0/95

All sequence variants present in the coding region of *SP4* were found in exon three. The only *SP4* variant that segregated with disease was Asn306Ser.

**Figure 1:**
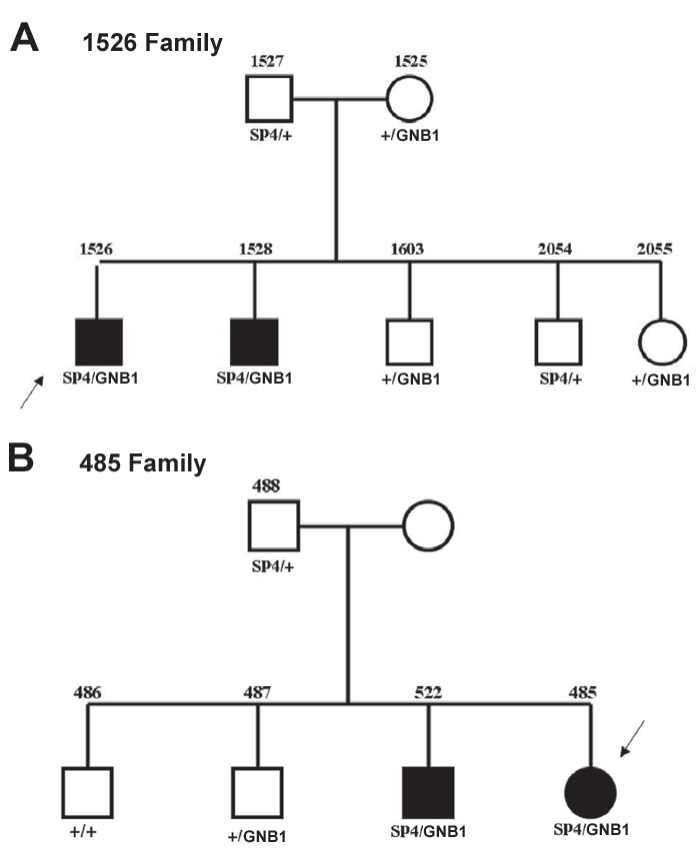
Pedigrees number 485 and number 1526 segregate the *SP4/GNB1* alleles. Filled symbols indicate individuals with retinitis pigmentosa. Probands are indicated by arrows. The allele designation *SP4* denotes the Asn306Ser allele, *GNB1* denotes the A-G substitution in intron 2 of the *GNB1* gene, + denotes normal alleles.

Table 3 shows the results of previous screenings of several photoreceptor genes in the six probands with the Asn306Ser mutation in one allele. Patient 856 has arCRD while the other 5 patients have arRP. Three of the six probands also carried an A-G variant in intron 2 of the *GNB1* gene. DNAs of the family members of one of these probands could not be obtained (family 2177). However, in the families of the other two probands, the *SP4* missense and the *GNB1* intronic variant segregated with disease (Figure 1A,B). None of the other variants in the screened genes segregated with disease. We found no additional variants in the genes encoding α-, β- and γ-cGMP-phosphodiesterase, RDS/peripherin or the β-subunit of transducin in the RP isolate patient homozygous for Asn306Ser.

**Table 3:** Sequence variants in five genes of six patients with the heterozygous *SP4* Asn306Ser mutation.

Patient	*PDE6A*	*PDE6B*	*PDE6G*	*GNB1*	*RDS*
449	none	none	none	none	none
485	Exon 14, Phe597Phe	none	none	Intron 2, A to G, 103rd bp from 3’ splice site	none
	Intron 18, A to C, 21st bp from 3’ splice site				
	Exon 20, Phe779Phe				
856	not done	not done	not done	none	not done
1526	Intron 18, A to C, 21st be from 3’ splice site	Exon 6, Ile330Ile	Intron 3, C to G, 9th bp of 3’ splice site	Intron 2, A to G, 103rd bp from 3’ splice site	none
	Exon 20, Phe779Phe				
1824	none	none	none	none	none
2177	none	none	none	Intron 2, A to G, 103 rdbp from 3’ splice site	none

The probands of three families (485, 1526, and 2177) had an intronic sequence variant in *GNB1* in addition to *SP4* Asn306Ser. These sequence variants segregated with disease in families 485 and 1526. We were unable to obtain the DNA of members of family 2177. The sequence variant in intron 18 of *PDE6A* did not segregate with disease in families 485 and 1526.

## Discussion

Even though 2/3 of mice born with the *Sp4* gene deleted die within the first four weeks of life [14], the surviving mice have many abnormalities including severe retinal degeneration (our data, not shown). Therefore, we considered the human *SP4* gene a good candidate for the site of missense mutations causing retinal degeneration, given its involvement in the transcription of several photoreceptor genes including *PDE6B* [13,15,16] and the phenotype of the *Sp4* knockout mouse.

We found nine unique sequence variants in the *SP4* gene of 29 patients affected with different types of inherited retinal degenerative disease. Of these, five variants were in one of the six exons of *SP4*. Two of the variants coded for the same amino acid (Ala276Ala and Gln451Gln). Two more variants predicted amino acid changes in the SP4 protein, Leu241Val and Pro286Ala, but neither segregated with disease in the corresponding families. The fifth missense Asn306Ser mutation was present in seven probands. One of the probands that had the homozygous Asn306Ser mutation was an isolate with RP, so we could not tell if the two Asn306Ser alleles were causing disease. In family 449, an affected sibling of the proband did not carry the Asn306Ser allele; for the probands of families 856 (arCRD) and 1824, neither a second variant *SP4* allele nor a variant in any of the other genes previously screened could be identified. The three remaining probands all carried Asn306Ser and an intronic A-G substitution 103 bp upstream of the 3' splice site of intron 2 of the *GNB1* gene. We could not obtain DNAs from the family of one of the probands (2177), but in the families of the other two probands (485 and 1526) only the two affecteds in each pedigree carried both alleles (Figure 1A,B). Both of these families had arRP. All seven families carrying Asn306Ser were of Hispanic background and so was the 1 control carrying the same mutation. Thus, 18.4% of the Hispanic patients carried this allele while none of the patients from other backgrounds did (0/151 Europeans, 0/35 Blacks and 0/46 Asians). The higher frequency of Asn306Ser in Hispanic patients (p<0.001 applying the Fisher's exact test) compared to a relatively low frequency in Hispanic controls (1/12=8.3%) suggests that this variant may be pathogenic.

**Figure 2:**
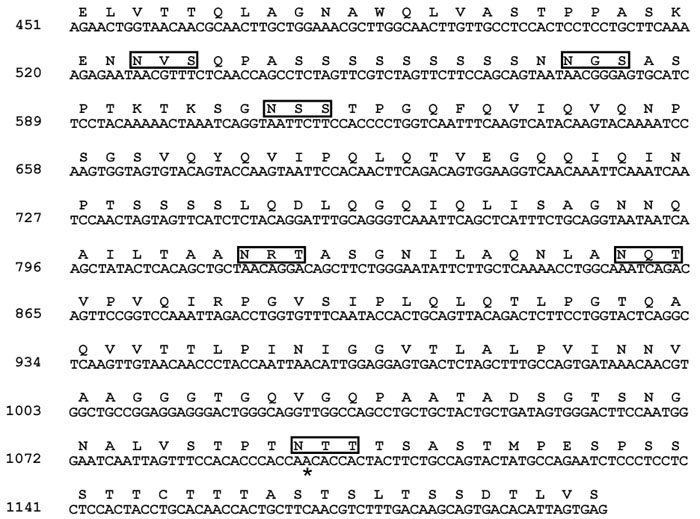
Partial nucleotide sequence of the cDNA encoding human *SP4* and the corresponding predicted amino acid sequence. Boxes indicate potential N-glycosylation sites (N-X-S/T). Asterisk (*) indicates the location of the A to G mutation (AAC to AGC causes the change of Asn306 to Ser).

There are several additional reasons that implicate *SP4* Asn306Ser as a mutant allele that may contribute to autosomal recessive disease. (1) We have no family history for the proband that carried two alleles of Asn306Ser. Therefore, the two Asn306Ser alleles together could be the cause of that isolate patient's disease. (2) In each of the three families where the Asn306Ser allele did not segregate with disease, two mutant alleles in other genes may have rendered the presence of the heterozygous Asn306Ser coincidental and unrelated to disease. However, this does not rule out the possibility that two Asn306Ser alleles could cause disease. (3) For the two families where only the affecteds carried both the Asn306Ser allele and the *GNB1* variant intronic allele, pathogenesis is possible at least genetically. Furthermore, *GNB1* has three GC boxes in its promoter and SP4 interacts with GC boxes. Thus, digenic disease may be plausible. To answer the question of pathogenicity of Asn306Ser, functional assays of the protein carrying this variant would have to be conducted. Nevertheless, the possibility that Asn306Ser may be pathogenic is supported by the fact that Asn306Ser is in the transactivation domain of the SP4 protein and in one of six glycosylation sites (N-X-S/T; Figure 2). Changing asparagine to serine eliminates this site of posttranslational modification and this may affect the function of the protein. Interestingly, at position 306 there is an asparagine only in the human SP4 sequence while in the mouse, rat, dog and cow there is a threonine. Therefore, asparagine is not a conserved residue. With regard to the *GNB1* intronic variant, it is not in a splice site or a consensus branch point, but it may be in a heretofore-unknown regulatory region.

For the intronic *GNB1* variant, a DNA fragment including exon 2, intron 2 (carrying the variant), and exon 3 would have to be expressed to determine if this variant caused a splicing problem. However, the sequences adjacent to the A-G substitution do not correspond to consensus branch point sequences. Another possibility is that the A-G substitution would disrupt an enhancer or a repressor sequence causing altered expression of the *GNB1* gene. Although there is no direct evidence that the Asn306Ser mutation in the *SP4* gene and the intron 2 A-G variant of the *GNB1*gene together are responsible for disease in the affected individuals, digenic disease cannot be ruled out without further testing the pathogenicity of the alleles. It is certainly plausible that the protein products of a phototransduction gene like *GNB1* and a transcription factor that may influence its expression like *SP4* can together cause digenic disease when one allele of each carries a mutation.
